# Strengthening National Public Health: first steps towards the Spanish Public Health Agency

**DOI:** 10.1093/eurpub/ckac131.541

**Published:** 2022-10-25

**Authors:** G Barbaglia, D García-Abiétar, JL Beltrán-Aguirre, AM García, S García-Armesto, I Gutiérrez-Ibarluzea, A Segura-Benedicto, M Franco-Tejero, R Urbanos-Garrido, I Hernández-Aguado

**Affiliations:** 1 Spanish Society of Public Health, Barcelona, Spain; 2 Preventive Medicine and Public Health, Hospital del Mar Teaching Unit, Barcelona, Spain; 3 Faculty of Law, Public University of Navarra, Pamplona, Spain; 4 Institute of Health Sciences in Aragon, Zaragoza, Spain; 5 Fund Vasca Innovación e Investigación Sanitar, BIOEF, Bizkaia, Spain; 6 Catalonia Bioethics Committee, Public Health Advisory Council, Catalonia, Spain; 7 John Hopkins University, Bloomberg School of Public Health, Baltimore, USA; 8 Public Health Department, Miguel Hernández University, Alicante, Spain; 9 Care and Prevention of Drugdependences, Public Health Agency, Barcelona, Spain; 10 Spanish Society of Public Health, Spain; 11 Department of Applied Economics, Universidad Complutense de Madrid, Madrid, Spain

## Abstract

**Issue/Problem:**

The social, ecological, health and economic crisis exacerbated by COVID-19 is a challenge of extraordinary magnitude and complexity for global public health. Part of the response to these challenges requires strong public health institutions.

**Description of the problem:**

Component 18 of the Government of Spain’s Recovery, Transformation and Resilience Plan proposes the creation of a Spanish Agency of Public Health (SAPH), a centre of excellence to perform surveillance and health system preparedness functions in the face of new or emerging public health threats. The government has opened the debate on what design and functions it should have.

**Results:**

SESPAS, the Spanish Society of Public Health and Public Administration, set out a proposal for the design and organization of the future Spanish Agency for Public Health [SAPH]. A large working group, made up of experts from various fields of public health and from its ten federated scientific societies, has designed the SAPH proposal. The scope of the public health functions to be assumed include: the strategic planning of the State’s public health (including health security), the prioritisation of public health policies and communication strategies, the assessment of the population’s health status and social conditions, the guarantee of the transversality and horizontality of health policies (health and health equity in all State policies), as well as their verticality (serving as an enriching link between the European Union level and the administrations of the Autonomous Communities and local authorities). The proposal also include 11 recommendations on the main elements to be taken into account in the establishment of the future SAPH.

**Lessons:**

COVID19 pandemic has highlighted the need of creating strong public health institutions. Spain is giving its first steps toward the creation of the SAPH, which constitute a key step in addressing the current and future challenges of public health in Spain.

**Key messages:**

• Spanish Public Health Agency is an opportunity to transform health policies and to improve the health of the Spanish population.

• Strengthening public health starts with building national institutions that ensure comprehensive and integrated health policies.

